# Breaking down the cell wall: Still an attractive antibacterial strategy

**DOI:** 10.3389/fmicb.2022.952633

**Published:** 2022-09-23

**Authors:** Jingxuan Zhou, Yi Cai, Ying Liu, Haoyue An, Kaihong Deng, Muhammad Awais Ashraf, Lili Zou, Jun Wang

**Affiliations:** ^1^The People’s Hospital of China Three Gorges University, Yichang, Hubei, China; ^2^Hubei Key Laboratory of Tumor Microenvironment and Immunotherapy, College of Basic Medical Sciences, China Three Gorges University, Yichang, Hubei, China; ^3^The Institute of Infection and Inflammation, College of Basic Medical Sciences, China Three Gorges University, Yichang, Hubei, China; ^4^Department of Microbiology, Government College University Faisalabad, Faisalabad, Pakistan

**Keywords:** peptidoglycan, lipopolysaccharide, teichoic acid, lipoprotein, antimicrobials

## Abstract

Since the advent of penicillin, humans have known about and explored the phenomenon of bacterial inhibition *via* antibiotics. However, with changes in the global environment and the abuse of antibiotics, resistance mechanisms have been selected in bacteria, presenting huge threats and challenges to the global medical and health system. Thus, the study and development of new antimicrobials is of unprecedented urgency and difficulty. Bacteria surround themselves with a cell wall to maintain cell rigidity and protect against environmental insults. Humans have taken advantage of antibiotics to target the bacterial cell wall, yielding some of the most widely used antibiotics to date. The cell wall is essential for bacterial growth and virulence but is absent from humans, remaining a high-priority target for antibiotic screening throughout the antibiotic era. Here, we review the extensively studied targets, i.e., MurA, MurB, MurC, MurD, MurE, MurF, Alr, Ddl, MurI, MurG, lipid A, and BamA in the cell wall, starting from the very beginning to the latest developments to elucidate antimicrobial screening. Furthermore, recent advances, including MraY and MsbA in peptidoglycan and lipopolysaccharide, and tagO, LtaS, LspA, Lgt, Lnt, Tol-Pal, MntC, and OspA in teichoic acid and lipoprotein, have also been profoundly discussed. The review further highlights that the application of new methods such as macromolecular labeling, compound libraries construction, and structure-based drug design will inspire researchers to screen ideal antibiotics.

## Introduction

An important feature that distinguishes bacteria from mammalian cells is the cell wall; its mechanical strength depends on the existence of peptidoglycan (PG), providing bacteria with a rigid structure (Sibinelli-Sousa et al., 2021). Lipopolysaccharides (LPS) is the main component of the outer membrane (OM) of gram-negative (G^−^) bacteria, and plays an important role in the generation of drug resistance, the transportation and folding of extracellular proteins, nutrient absorption, and various signal transductions (Kutschera and Ranf, 2019; Simpson and Trent, 2019). Teichoic acid (TA) is an anionic carbohydrate polymer that accounts for 30 to 60% of the dry weight of the cell wall in gram-positive (G^+^) bacteria (Wu et al., 2021). According to the different binding sites, it can be divided into wall teichoic acid (WTA) that is bound to peptidoglycan n-n acetyl muramic acid (N-acetyl muramic acid) residue through phosphodiester bond and lipoteichoic acid (LTA) embedded in cell membrane through lipid anchoring (Wu et al., 2021). Different G^+^ bacteria have species-or strain-specificity in the WTA (Brown et al., 2010). Lipoproteins are lipid-anchored proteins that play an important role in obtaining nutrients and certain ions, the generation of drug resistance, and the transport and folding of extracellular proteins (El Rayes et al., 2021).

The complex polymers listed above that comprise the cell wall provide bacteria with strength and a barrier to the outside world, allowing them to thrive in a multitude of environments, including the human body (Brown et al., 2020). With the announcement that many major pharmaceutical companies will no longer fund research programs in this critical area, the influential and conscientious researchers need to identify new opportunities to combat the situation (Hu, 2018). Meanwhile, as a strategic object of research, the bacterial cell wall remains at the core of experimental practices, scientific narratives, and research funding appeals throughout the antibiotic era. Thus, the research laboratory was dedicated to the screening of new antibiotics while remaining the site at which the mode of action of new substances was investigated.

This article reviews and summarizes the iterative studies of the bacterial cell wall and the corresponding antibiotics to promote both mechanistic insights and translational applications, laying a solid foundation for finding more effective antibiotics.

## Peptidoglycan

PG is an important component of the cell wall that provides bacteria with a rigid structure and enables them to survive in hypotonic environments, which makes it a reliable target for antibiotic screening.

### The peptidoglycan synthesis

**Figure 1:**
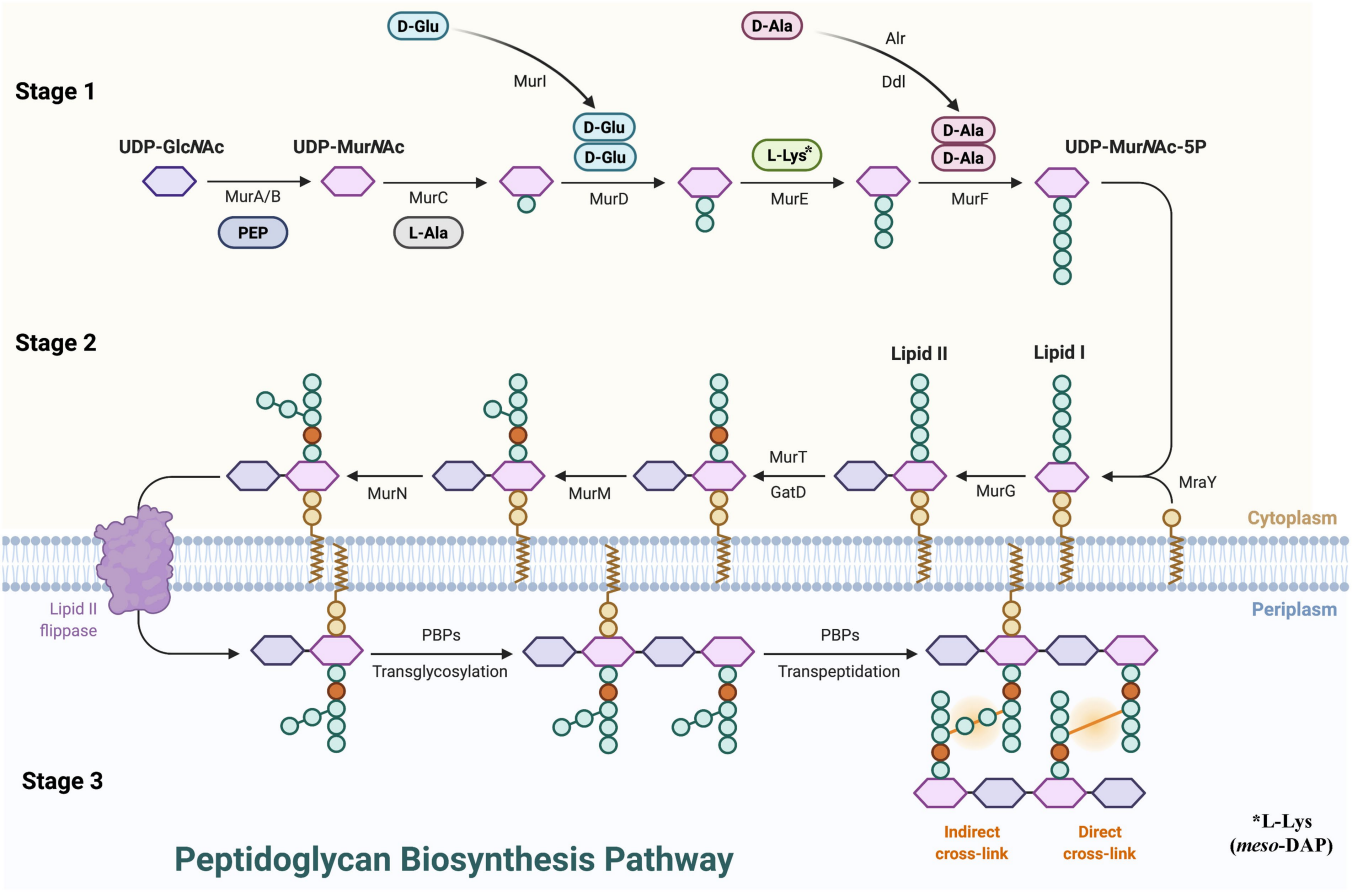
Peptidoglycan biosynthesis pathway. UDP-GlcNAc, uridine diphosphate N-acetylglucosamine; PEP, phosphoenol pyruvate; MurA, UDP-GlcNAc enol pyruvate transferase; MurB, UDP-N-acetyl allylacetone glucosamine reductase; MurG, decadecenyl diphosphate-MurNAc-pentapeptide-UDP-GlcNAcGlcNAc transferase; MraY, UDP-MurNAc-pentapeptide phosphotransferase; PBP, penicillin-binding proteins.

PG synthesis can be roughly divided into three stages (Figure 1). (1) Stage 1, in the cytoplasm, the PG precursor UDP-MurNAc-pentapeptide is formed. N-acetyl-L-phosphate glucosamine uracil transferase (GlmU) catalysis was used to produce uridine diphosphate N-acetylglucosamine (UDP-GlcNAc) from glucosamine-1-phosphate. The enol pyruvate group is then transferred from phosphoenol pyruvate (PEP) to the 3′-hydroxyl of UDP-GlcNAc by UDP-GlcNAc enol pyruvate transferase (MurA) to form UDP-GlcNAc-enol pyruvate (Wanke and Amrhein, 1993). Then UDP-GlcNAc is enolized and reduced to UDP-MurNAc by UDP-N-acetyl allylacetone glucosamine reductase (MurB) catalysis (Benson et al., 1993). L-Ala, D-Glu, m-DAP, or L-Lys, and D-Ala are subsequently treated by a series of ligases (MurC, MurD, MurE, MurF), and D-Ala forms UDP-MurNAc-pentapeptide with the participation of ATP (Green, 2002). (2) Stage 2, at the membrane, the assembly of the precursor to form lipid I and lipid II on the cytoplasmic side of the cell membrane. Phospho-MurNAc-pentapeptide was transferred from UDP-MurNAc-pentapeptide to undecaprenyl phosphate by UDP-MurNAc-pentapeptide phosphotransferase (MraY) catalysis to form lipid I, and GlcNAc combines with lipid I to form lipid II *via* decadecenyl diphosphate-MurNAc-pentapeptide-UDP-GlcNAcGlcNAc transferase (MurG; Anderson et al., 1967). After lipid II assembly is completed, the intermediate is flipped from the cytoplasmic surface of the membrane to the external surface which is mediated by SEDS (shape, extension, division, and spore formation) proteins or MOP (Multi-drug/oligosaccharide-lipid/polysaccharide) outputs (van Dam et al., 2007; Mohammadi et al., 2011; Ruiz, 2015). (3) Stage 3, polymerization and cross-linking of PG occurs on the surface of the cell membrane. MurJ and Class I PBPs transfer class II lipids through the cytoplasmic membrane, resulting in polymerization of glycan components. In addition, Class II PBPs catalyze crosslinking between adjacent stem peptides and then hydrolyze UndPP by undecaprenyl pyrophosphate phosphatase(s) to regenerate UndP (Goffin and Ghuysen, 1998; Ruiz, 2008). The lipid-linked disaccharide pentapeptide is converted into PG through transglycosylation and transpeptide to complete peptide cross-linking, which maintains the shape of the bacteria and guarantees the toughness of the PG.

**Figure 2:**
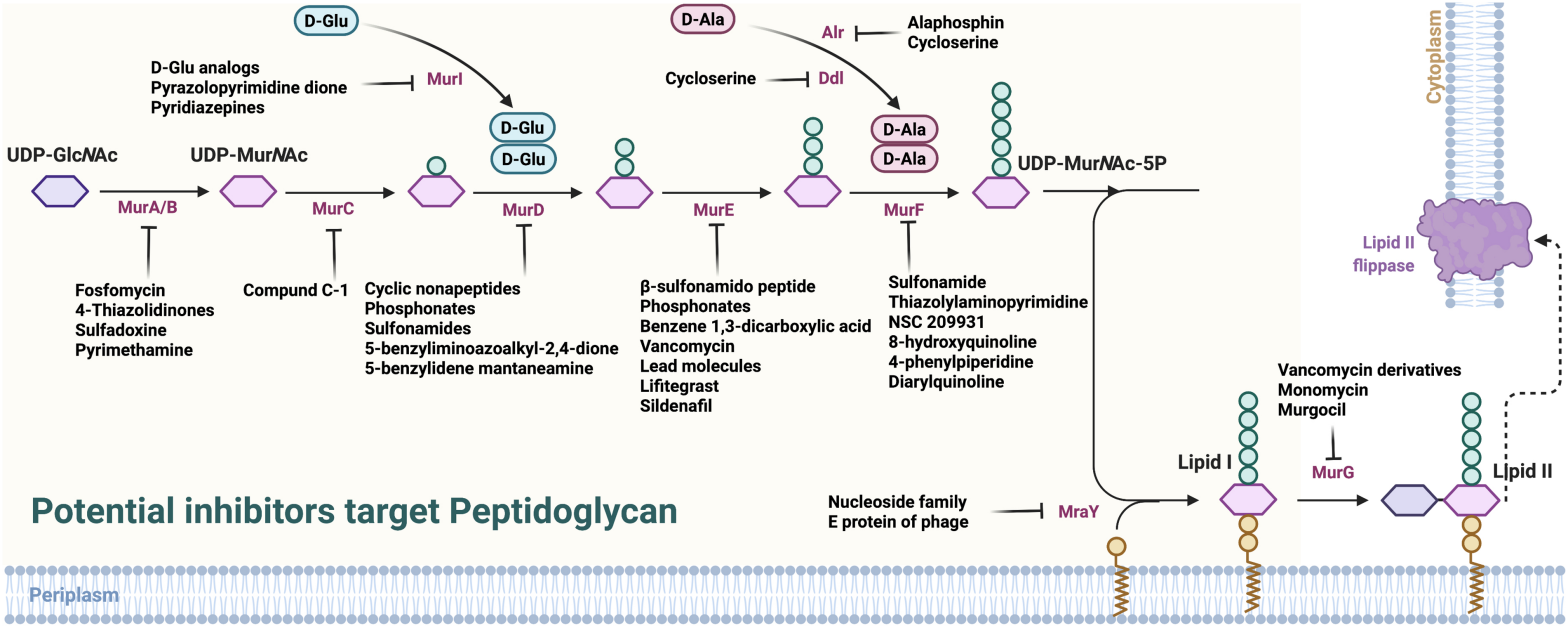
Potential inhibitors target peptidoglycan. UDP-GlcNAc, uridine diphosphate N-acetylglucosamine; PEP, phosphoenol pyruvate; MurA, UDP-GlcNAc enol pyruvate transferase; MurB, UDP-N-acetyl allylacetone glucosamine reductase; MurG, decadecenyl diphosphate-MurNAc-pentapeptide- UDP-GlcNAcGlcNAc transferase; MraY, UDP-MurNAc-pentapeptide phosphotransferase; PBP, penicillin-binding proteins.

Considering the key enzymes in PG synthesis, some potentially feasible candidates have been found to inhibit its synthesis (Figure 2; Supplementary Table S1).

### MurA

As the first key enzyme in catalyzing PG synthesis, MurA has been conducted to screen or design developable inhibitors (Tiwari et al., 2021). Fosfomycin has been discovered as the first irreversible inhibitor of MurA by the phenotypic screening (Diez-Aguilar and Canton, 2019), which blocks the PEP site by alkylating the thiol of Cys^115^, thereby blocking the PEP’s connection to the 3′-hydroxyl group of UDP-GlcNAc. In addition, Fosfomycin can enter bacteria through two uptake routes, namely L-α-glycerophosphate and hexosan-6-phosphate transporter systems, facilitating their functioning, at least in *Escherichia coli* (*E. coli*). Finally, Fosfomycin inhibits platelet activator receptors in respiratory epithelial cells, thereby reducing the adhesion of *Streptococcus pneumoniae* and *Haemophilus influenzae* (Wanke and Amrhein, 1993; Falagas et al., 2016). Further, it has been successfully used to treat urinary tract infections caused by *E. coli*, *Pseudomonas aeruginosa* (*P. aeruginosa*), and vancomycin-resistant *Enterococcus faecalis* (Lopez-Montesinos and Horcajada, 2019; Zhanel et al., 2020). Although new MurA inhibitors have been persistently discovered by screening, not all of them can covalently and irreversibly bind to MurA like Fosfomycin, i.e., RWJ-3981, RWJ-140998, RWJ-110192 (Baum et al., 2001). Through macromolecular labeling, it has been found that the above inhibitors do not promote the uptake of propidium iodide, thus the antibacterial effect may not be related to the cell damage (Silver, 2013). In recent years, more MurA inhibitors, including IN00152, IN00156, allylpyrocatechol derivatives, and heterocyclic electrophiles, have been discovered (Keeley et al., 2018;Kurnia et al., 2020; Raina et al., 2021), which highlighted the involvement of computer simulation and screening in future research. Among these, IN00152, IN00156, and allylpyrocatechol derivatives can non-competitively inhibit substrates UDP-GlcNAc and PEP, and the effectiveness increases along with the substrate concentrations (Kurnia et al., 2020; Raina et al., 2021). Furthermore, by combining IN00152 and IN00156 with antibiotics (carbenicillin, ciprofloxacin, gentamicin, and tetracycline), the additive interaction between them has been elucidated (Raina et al., 2021).

### MurB

In the synthesis progress of PG, MurB reduces the UDP-GlcNAc enol to UDP-MurNAc, which is a nucleotide sugar attached to the pentapeptide stem and a strong feedback inhibitor of MurA. Thus, MurB inhibitors can completely prevent inhibitory feedback from MurA. As Sulfadoxine (−7.3 kcal/mol) and Pyrimethamine (−7.8 kcal/mol), they possessed stable interactions with MurB of *M. tuberculosis* (Rani et al., 2020). Subsequently, purine-connected piperazine derivatives were synthesized, which had an inhibitory effect on *Mycobacterium tuberculosis* H37Rv (Konduri et al., 2020). A study has shown that 4-thiazolidinone is an inhibitor of diphosphate mimics with no antibacterial activity, but its core could be modify to Imidazolinone to have an effect (Andres et al., 2000). However, apart from its correlation with structure, there is no direct evidence showing the antibacterial activity depends on its inhibition of MurB (Bronson et al., 2003). As inhibitors of MurB, 3,5-Dioxopyrazolidines have antimicrobial activities against methicillin-resistant *Staphylococcus aureus* (MRSA), vancomycin-resistant *Enterococcus*, and penicillin-resistant *Streptococcus pneumoniae*, which also exhibits inhibition effects on MurA and/or MurC (Yang et al., 2006). However, since serum might bind to 3,5-Dioxopyrazolidines to reduce the antibacterial activity by influencing their interaction with the bacterial targets (Merrikin et al., 1983), there are difficulties in evaluating the efficacy and safety of 3,5-Dioxopyrazolidines-derived compounds *in vivo*. For *P. aeruginosa*, fragments of pyrazole derivatives were identified through differential scanning fluorimetry and X-ray crystallography, which were synthetically modified to enhance their binding affinities. Based on those fragments, the inhibitors of MurB can be designed in the future against *P. aeruginosa* (Acebron-Garcia-de-Eulate et al., 2022). Furthermore, molecular docking suggested that the S atom of thiazole in the sulfur ether part is connected to the amino acid residue in the MurB enzyme by hydrogen bonding, which implied that by increasing the sulfur ether part could assist new MurB inhibitors screening (Sanad et al., 2020). In addition, studies showed that thiazolyl urea-like structures can inhibit bacterial respiration, but the mechanism remains unclear (Proctor et al., 2002; Francisco et al., 2004).

### MurC

MurC is responsible for adding the L-alanine (L-Ala) to the nucleotide precursor UDP-MurNAc, and its inhibitors usually competitively inhibit the MurNAc binding site. For example, Compound C-1 shares similar ATP binding sites as MurC; it can inhibit MurC of *E. coli* and those intestinal bacteria that are closely related to *E. coli*. Although *H. influenzae* shares the highly conserved ATP binding region of MurC with *E. coli*, Compound C-1 showed no effect on *Haemophilus* MurC and the growth of other G-bacteria (Zawadzke et al., 2008). Therefore, the development of MurC inhibitors still needs further exploration. In addition, MurC and MurD shares a similar binding site as the UDP-Nacetylmuramyl moiety. Meanwhile, MurE and MurF possess different binding sites. This property might direct the inhibitors’ screening direction.

### MurD

MurD is responsible for adding ATP-dependent D-glutamic acid to UDP-MurNAc-L-Ala and is involved in acylphosphate and tetrahedral intermediates in PG synthesis. MurD inhibitors can be roughly divided into two types: peptides and non-peptides. Two cyclic none-residue peptides were found to inhibit MurD of *E. coli*, i.e., Cys-Pro-Ala-His-Trp-Pro-His-Pro-Cys and Cys-Ser-Ala-Trp-Ser-Asn-Lys-Phe-Cys, with 1.5 and 0.62 mmol/l as IC_50_ values, respectively (Bratkovic et al., 2008).

Non-peptide inhibitors can be further divided into two categories according to whether they are based on glutamate or not, and glutamate-based inhibitors account for the vast majority, including the following subcategories: Phosphonates, Sulfonamides, 5-benzyliminoazoalkyl-2, 4-dione/5-benzylidene mantaneamine, and 5-benzylidenethiazolidin-4-one. Phosphonate transition state analogs are the earliest-found MurD inhibitors. The MurD of *E. coli* can be inhibited by replacing the MurNAc part with a suitable length of hydrophobic linker (Tanner et al., 1996). Sulfonamides are another important class of MurD inhibitors, which are like Phosphonates in that their amide functional group is incorporated into the target compound to simulate MurD tetrahedral intermediates (Kotnik et al., 2007; Humljan et al., 2008). Its L-and D-glutamic acid components are in the same position as the glutamic acid component of UDP-MurNAc-L-Ala-D-Glu, and its inhibitory effect is competitive with that of D-glutamic acid. 5-benzyliminoazoalkyl-2,4-dione and 5-benzylidene amantadine, which are MurD inhibitors, still possess activities after replacing D-Glu with L-Glu (Tomasic et al., 2009). The inhibition effect even become better through a slight change of the ring substituent and the linker region between the two benzene rings (Tomasic et al., 2012). The most powerful change is achieved by designing and synthetizing a NH-CH_2_ group to connect the two inverted benzene ring compounds, which can greatly increase hydrophobic interactions and hydrogen bonds through water molecules. It can also inhibit MurE as well and showed antibacterial activity against MRSA (MIC, 8 μg/ml; Tomasic et al., 2012). 5-benzylidenethiazolidin-4-one can inhibit MurD, based on which several different compounds were designed and optimized. These compounds showed strong inhibitory activities against *E. coli* MurD ligase with an IC_50_ of ~28 mM (Zidar et al., 2011). In addition, some compounds also showed antibacterial activities against G^+^ bacteria including *S. aureus* and *Enterococcus faecalis*. In addition, studies on X-ray crystal structure reveals the structure–activity relationship, showing a new direction on the design and development of new inhibitors of MurD (Zidar et al., 2011). Although many types of *E. coli* MurD inhibitors have been discovered, MurD orthologs from other pathogens, such as *S. aureus*, showed low sensitivity to some types of inhibitors (Barreteau et al., 2012). The overall similarity of the amino acid sequences of MurDs in different bacteria was low, while sharing conserved residues closely related to catalytic activity. This low sensitivity may be related to the slow growth characteristics of bacterial species, which suggest the difference in active site topology plays a crucial role in the identification process (Barreteau et al., 2012). Therefore, the structure of MurD orthologs must be taken into consideration for screening better MurD inhibitors.

### MurE

MurE is responsible for linking L-lys or mDAP to UDP-MurNAc-L-Ala-D-Glu, which plays an important role in stemming the peptide in the third position. Since MurE follows MurD in the cascade of amino acid additions in PG synthesis, there is a similar structure between the transition state analogue inhibitor of MurD and the substrate of MurE. β-sulfonamido peptide is a moderate inhibitor of *S. aureus* MurE (El Zoeiby et al., 2003). The phosphonates that inhibit MurD also act as inhibitors of MurE. As a benzene, 1,3-dicarboxylic acid can dually inhibit MurD and MurE through the modification of certain chemical groups (Perdih et al., 2009). By virtual screening, molecular dynamics, and *in vitro* studies, some researchers have found that Lead molecules can effectively inhibit the growth of *S. aureus,* and the activity is related to the exposure-response relationship. The bactericidal effect of a Lead molecule works quite similarly to Vancomycin, both of which can inhibit the growth of *S. aureus* in a dose-dependent manner (Zaveri and Kiranmayi, 2017). Vancomycin acts on the D-Ala-D-Ala part of the NAM and NAG cross-linked on the cell surface, while the Lead molecule targets the cytoplasmic MurE. Because of the difference in targets, Lead molecules are used as new antibiotics to replace Vancomycin to deal with the clinical Vancomycin-resistant bacteria, including Vancomycin-resistant *S. aureus* (VRSA), Vancomycin intermediate *S. aureus* (VISA), and heterogeneous Vancomycin-intermediate *S. aureus* (hVISA; Appelbaum, 2007). Recent studies showed that Lifitegrast (−10.5 kcal/mol) and Sildenafil (−9.1 kcal/mol), which have been approved by the FDA, can combat MurE of *Mycobacteria* spp. (Rani et al., 2019), which provides a new research direction for tuberculosis treatment.

### MurF

MurF catalyzed the addition of D-Ala-D-Ala to the nucleotide precursor UDP-MurNAc-L-Ala-G-D-Glu-meso-diaminopimelate (UMtri-mDAP). Transition state Pseudotripeptide and Pseudotetrapeptide aminoalkyl phosphinate mimetics were the earliest reported MurF inhibitors. Subsequently, Sulfonamide inhibitors were discovered and could be crystallized together with MurF (Longenecker et al., 2005). Later, Thiazolylaminopyrimidine series were identified as the MurF inhibitors, of which the lowest IC_50_ was 2.5 μM (Baum et al., 2006). However, none of the inhibitors had antibacterial activity, which may be related to their poor permeability of the cell wall (Baum et al., 2009). Based on the release of the crystal structure of *Streptococcus pneumoniae* MurF, a new MurF inhibitor, NSC 209931, was screened virtually, with IC_50_ of 63 μM and MIC of 128 μg/ml, respectively (Turk et al., 2009). In addition, the series of 8-hydroxyquinoline, 4-phenylpiperidine derivatives, and Diarylquinoline can inhibit MurF of *E. coli* (Baum et al., 2007, 2009). Furthermore, based on the previously released inhibitors, nanomolar inhibitors of *S. pneumoniae* MurF (MurFSp), micromolar inhibitors of *E. coli* (MurFEc) and *S. aureus* (MurFSa) have been modified by Lead compounds to produce new effective inhibitors (Hrast et al., 2013). The discovery and evolution of these inhibitors provide a new spark for creating novel antibacterial candidates.

### Alr and Ddl

Alr (alanine racemase) and Ddl (D-Ala-D-Ala ligase) are enzymes required to produce D-Ala from L-Ala and link two D-Ala parts. In *M. tuberculosis*, Alr and Ddl are both essential and are inhibited by Cycloserine (Sassetti et al., 2003; Bruning et al., 2011), the second line of antituberculosis drugs. Although the main target of Cycloserine in *M. tuberculosis* remains controversial, the results showed that both required targets can be inhibited by a single entity (Feng and Barletta, 2003; Bruning et al., 2011). In *Streptococcus* spp., Cycloserine has been shown to inhibit Alr and Ddl *in vitro* through mutations (Reitz et al., 1967; Noda et al., 2004). In addition, Roche designed a synthetic Alr inhibitor, Alaphosphin, which is taken up by peptide transport. However, the loss of permease (in this case, tripeptide permease, tpp) leads to drug resistance (Atherton et al., 1979).

### MurI

MurI is a glutamate racemase that produces enough D-Glu to be linked to UNAM-L-Ala through MurD. The structure and function of MurI are regulated by a variety of factors. D-Glu transferase cannot be replaced in *S. aureus* and *S. pneumoniae*, since MurI is essential (Zhang et al., 2004). In *Listeria spp.* and *Bacillus spp.*, there is another enzyme, pyridoxal 5 phosphate aminotransferase, that can produce D-Glu (Barreteau et al., 2008), and there are two unique biosynthetic pathways for D-Glu in *Staphylococcus hemolyticus* (Pucci et al., 1995). A study showed that D-Glu analogues can inhibit MurI of *S. pneumoniae* with a MIC of 0.2 μg/ml, and many of them possess good correlations between enzyme inhibition and the MIC (de Dios et al., 2002). Researchers have found that Pyrazolopyrimidine dione (Lundqvist et al., 2007) and Pyridiazepines (Geng et al., 2009) have anti-*H. pylori* effects, which can interact with the allosteric active site of MurI. They can reduce the amount of D-Glu in the glutamate pool when combined with serum proteins. However, the two compounds have a high drug resistance property and showed no protective effect in the *H. pylori*-infected mouse model. Therefore, the pharmacokinetic characteristics of the two compounds need to be further studied, and the issue of drug resistance also needs to be taken into consideration (Lundqvist et al., 2007; de Jonge et al., 2009; Geng et al., 2009).

### MraY

Phosphate-MurNAc-pentapeptide transpotentator (MraY) is involved in the first step of catalyzing the lipid lipoligacy. It transfers phospho-MurNAc-pentapeptides into undecyl phosphate with the participation of Mg^2+^, producing undecylene-P–P-MurNAc-pentapeptide (lipid intermediate I) and uridine monophosphate (UMP). Members of the natural nucleoside antibacterial family (Tunicamycin, Moreomycin, Carpramycin, etc.), the antibiotic E protein of phage φX174, and some small-molecule compounds can all effectively inhibit MraY, exhibiting antibacterial activities.

Compounds in the nucleoside antibiotic family can recognize and competitively bind to the UDP-N-acetylmuramyl-pentapeptide (UDP-Mpp) binding site of MraY. These molecules all contain the same aminoribosyl-O-uridine backbone, which is very important for their inhibitory effects (Dini et al., 2000; Bugg et al., 2006; Tanino et al., 2011; Rodolis et al., 2014a,b). Although it can combine with the uracil part of the nucleoside inhibitor, uridine itself cannot act as an inhibitor of MraY. We can target the medicinal hot spots (the uridine adjacent, TM9b/Loop E, Caprolactam, Hydrophobic, Mg^2+^ cofactor, and Tunicamycin binding pockets) near the uridine binding site on the cytoplasmic surface to inhibit MraY (Mashalidis et al., 2019). The antibiotic E protein of phage φX174 affects the function of MraY in *E. coli* and blocks the PG synthesis; however, this phenomenon does not occur in G^+^ bacteria. By gene analysis, it was found that 29 amino acids from the N-terminal constitute the transmembrane domain of protein E. When the N-terminal rather than C-terminal hydrophobic residue is replaced by leucine, it loses its bioactivity. Thus, 29 amino acids from the N-terminal constitute the key sequence for the inhibition of MraY. Furthermore, by site-directed mutagenesis, researchers found out that the proline at position 21 in the transmembrane domain of protein E is also crucial for the inhibition of MarY (Witte et al., 1990; Bernhardt et al., 2001; Mendel et al., 2006; Zheng et al., 2008; Tanaka and Clemons Jr., 2012). Through the study of *Bacillus subtilis*, Chen et al. (2016) found that the first two amino acids of the Park’s nucleotide oligopeptide chain are very important for recognition of MraY. Thus, Park’s nucleotides, their analogs, and the C4-OH configuration play important roles in substrate specificity. Further studies discovered that modification at the 5-position of uracil can severely damage the activity of its substrate, which provides a direction for the design and development of MraY inhibitors. At present, significant progress has been made in the development of detection methods suitable for high-throughput screening. Researchers have been able to screen low-molecular-weight MraY inhibitors so far (Fer et al., 2018).

### MurG

MurG belongs to the glycosyltransferase family and connects the GlcNAc of UDP-GlcNAc to lipid I after MraY catalysis and lipid II production (Chen et al., 2002). There are three main methods for MurG inhibitors screening currently. The first one is to use existing inhibitors to synthesize similarly designed molecules. The second method is to synthesize UDP-GlcNAc mimics by modifying the nucleotide groups. The last one is to construct compound libraries and use purified enzymes to screen lead compounds that competitively bind to MurG (Helm et al., 2003; Hu et al., 2003, 2004).

Through the synthesis and determination of MurG transition state analogues, Amy et al. (Trunkfield et al., 2010) prepared a library of 19 analogues of *E. coli* MurG, most of which can inhibit MurG that contain a 2-methoxyphenyl R1 substituent. *In vitro*, Vancomycin derivatives containing N-chlorobiphenyl-N-methylleucine and Monomycin were reported as the effective inhibitors of MurG (Terrak et al., 1999; Liu et al., 2003), but none of them could penetrate the cell wall, nor could they exert MurG-inhibiting activity *in vivo*. As a newly discovered steroid-like molecule, Murgocil can also specifically bind to MurG, which can effectively inhibit the PG synthesis in *S. aureus* and has synergistic activity with β-lactam. In addition, studies have proved that the synergistic activity of Murgocil and Imipenem is mediated by the localization of MurG-dependent PBP2 in the division interval. Through the study of several murgocil-resistant *Staphylococci*, the possible sites of its resistance were found, which depends on certain unique amino acid residues in *S. aureus* MurG, unfortunately. Thus, the antibacterial activity of Murgocil in the body is limited to *Staphylococcus* spp.; it is ineffective against other G^+^ and G-bacteria (Mann et al., 2013). Therefore, the discovery of MurG inhibitors still needs further exploration.

## Lipopolysaccharide

**Figure 3:**
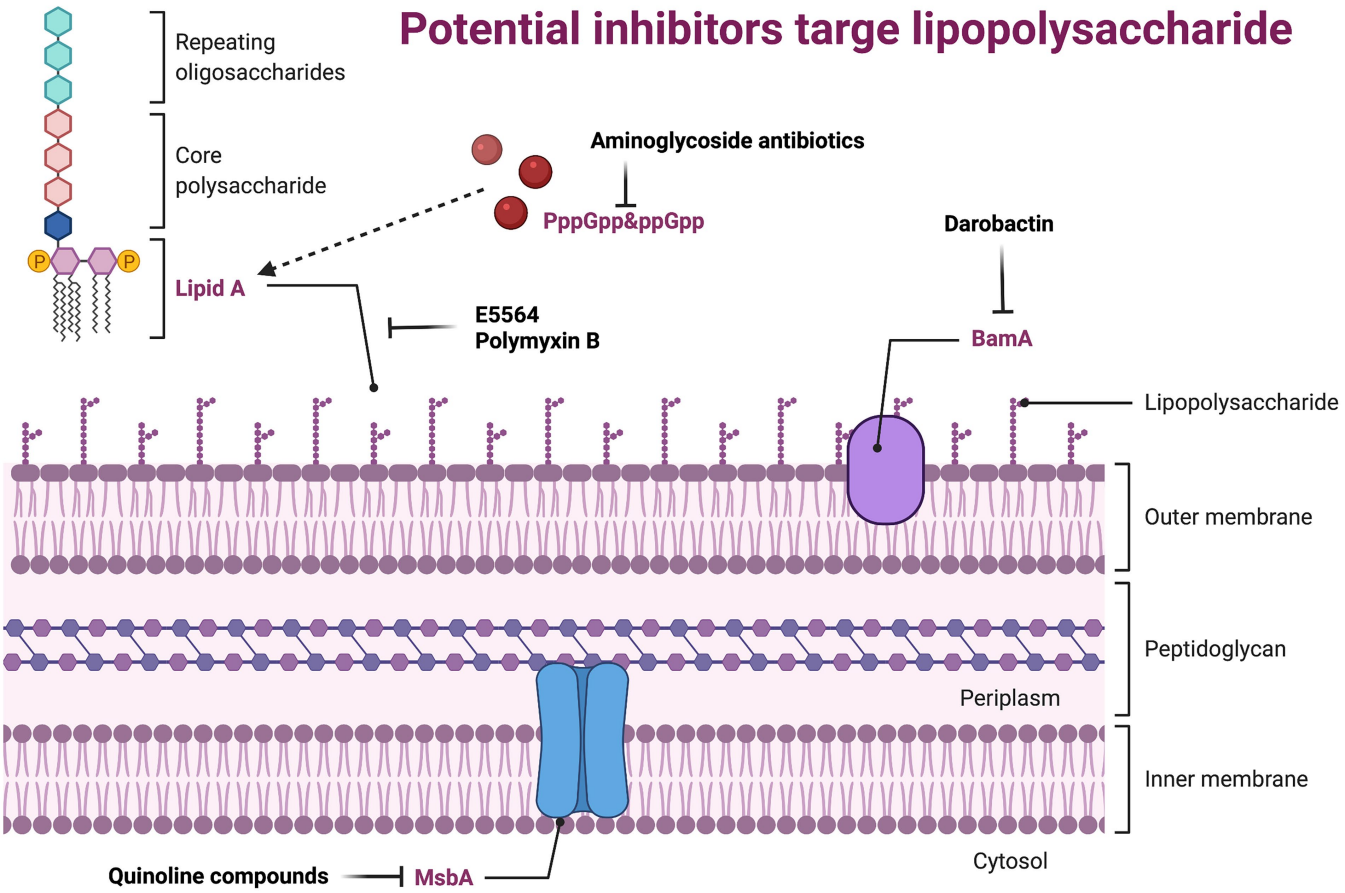
Potential inhibitors target lipopolysaccharide. BamA, the central component of the BamABCDE complex, it catalyzes both folding and insertion of nascent porins; MsbA, belongs to the ABC transporter superfamily, which reversely transports LPS from the intima leaflet synthesis site to the intima outer leaflet.

Lipopolysaccharide (LPS), also known as endotoxin, is the main component of the outer membrane (OM) of all G-bacteria. It maintains the structural integrity of the bacteria and protects the bacteria membrane from attack by certain chemicals. However, it is also the cause of various biological effects associated with G-sepsis. LPS can alter the morphology, metabolism, and gene expression of nearly all eukaryotic cells, as well as stimulate the uncontrolled expression of host cytokines and cause severe infection (Kutschera and Ranf, 2019; Simpson and Trent, 2019). LPS consists of three parts: lipid A, core polysaccharide, and O-specific polysaccharide. Lipid A is the basis for its production of toxic substances (Kawahara, 2021), core polysaccharide is composed of heptose, galactose, and 2-keto-3-deoxyoctanoic acid, and O-specific polysaccharide is a polymer formed by end-to-end repeating units of a specific length. G-bacteria’s membrane barriers include a phospholipid inner membrane and an asymmetric OM that is primarily composed of LPS (Osborn et al., 1972a,b; Kamio and Nikaido, 1976). Although membrane proteins and fibrous structures such as pili and flagella also help maintain the morphology of the bacterial membrane surface, the change of OM is lethal to bacteria (Lu et al., 2014). Therefore, developing new antibacterial agents targeting LPS is a highly strategy (Figure 3; Supplementary Table S2).

Uridine diphosphate-(3-O-(R-3-hydroxydanoyl))-N-acetylglucosamine deacetylase (LpxC) is a cytosolic zinc-based deacetylatase that catalyzes the first step of lipid A biosynthesis and is a validated antibiotic target. (Wyckoff et al., 1998). Therefore, inhibiting LpxC can interfere with the membrane stability of G-bacteria, such as how 2-(1Shydroxyethyl) imidazole achieves inhibition of LpxC through the imidazoly chelated zinc ion domain, with 4 μg/ml as the MIC against *P. aeruginosa* (Yamada et al., 2020). The most thoroughly studied inhibitor of LpxC is CHIR-090, which has inhibitory effects on most G-bacteria (Zhang et al., 2012).

In the host, cluster of differentiation (CD) 14 (Ulevitch and Tobias, 1995) and Toll-like receptor (TLR)-4/Myeloid differentiation (MD) 2 complex (Kim et al., 2007; Ohto et al., 2007; Zimmer et al., 2007) are responsible for the recognition of LPS. CD14 presents LPS to TLR-4/MD2 to trigger the synthesis of various inflammatory mediators, such as interleukin (IL)-1, IL-6, and tumor necrosis factor-α (TNF-α), and the production of costimulatory molecules that are needed to activate the adaptive immune response (Raetz and Whitfield, 2002; Brandenburg and Wiese, 2004; Miyake, 2004; Munford and Varley, 2006). During the whole course, the interaction between lipid A and TLR-4/MD2 is very critical. There is a study that shows that natural or synthetic lipid A derivatives, such as E5564 (Lynn et al., 2003), can be used as an antagonist of site-specific competition for lipid A receptors on the TLR-4/MD2 complex. LPS not only plays an important role in the TLR4-MD2 pathway, but also participates in pathogen-related molecular patterns (Rietschel et al., 1994; Snyder et al., 1999), and can help antimicrobial peptides or complement (Raetz, 1990). These are closely related to the immune response, which shows the feasibility and superiority of LPS as a new antibiotic.

Polymyxin B is a lipopeptide antibiotic isolated from *Bacillus polymyxa* (Storm et al., 1977), which can interact with the LPS through electrostatic force by its polycationic peptide ring. When polymyxin are close to OM, the branched alkyl tail of Polymyxin B will be drawn into the hydrophobic core of the OM to solubilize the inner membrane (Oh et al., 2017). Like Polymyxin, Darobactin, discovered in recent years, may also act on LPS. Darobactin is a heptapeptide that has antibacterial activity against a series of G-bacteria, including drug-resistant pathogens (Imai et al., 2019). Intriguingly, by sequencing, BamA was confirmed as the only OM protein that causes Darobactin resistance (Imai et al., 2019), and the direct inhibitory effect of Darobactin on BamA was further observed through *in vitro* protein renaturation experiments (Imai et al., 2019). Furthermore, the inhibition of Darobactin on BamA and the destruction of the formation of the OM is consistent. In addition to inhibiting the synthesis of bacterial proteins, the aminoglycoside antibiotics, including Gentamicin, Tobramycin, and Amikacin, can also inhibit the synthesis of LPS (Davis, 1987). The underlying mechanism may be related to inhibiting the accumulation of guanosine 5′-triphosphate-3′-diphosphate (PppGpp) and guanosine 5′-diphosphate 3′-diphosphate (ppGpp; Cortay and Cozzone, 1983).

The inner membrane ABC transporter MsbA belongs to the ABC transporter superfamily, which reversely transports LPS from the intima leaflet synthesis site to the intima outer leaflet (Doerrler et al., 2004; Ward et al., 2007). Studies have found that MsbA utilizes the ATP to bind and hydrolyze through an open inward (cytoplasmic) and open outward (periplasmic) conformation during the LPS transport cycle (Mi et al., 2017; Ho et al., 2018). Mutations that prevent conformational changes in the transmembrane domain can disrupt the hydrolysis of ATP (Doshi et al., 2013). Thus, molecules that can inhibit the conformational changes required for MsbA transport activity may also inhibit ATPase activity. Many Quinoline compounds (such as G592, G913, and G332 (Alexander et al., 2018)) with bactericidal activity were screened and were proven to selectively inhibit MsbA. Recently, researchers have discovered that certain compounds can stimulate the ATPase activity of MsbA while separating it from the LPS translocation, thereby interrupting the transport function (Zhang et al., 2018). This mechanism shares a similar phenomenon as inhibitors of ABC transporters. For example, G0507 (Nickerson et al., 2018) inhibits LolCDE (a protein that ensures the normal transport of lipoproteins from the inner membrane to the outer membrane) and tariquidar (Loo and Clarke, 2014) inhibits P-glycoprotein (an excretion pump in mammalian cells that expels the drug). In addition, MsbA inhibitors were found to be more effective against strains lacking TolC efflux pumps (Alexander et al., 2018). In general, the study of MsbA provides a leading basis for the development of new antibiotics.

## Teichoic acid

Teichoic acid is an important component of the vast majority of G^+^ bacterial cell walls. It is a polymer composed of polyphosphate glycerides or polyphosphate ribositol that plays an important role in maintaining ionic homeostasis in the cell wall, protecting bacteria from antibiotic and lysozyme damage, division and autolysis, and recognition and interaction with host cells. Glycosylated WTA plays an important role in cell morphology, bacterial colonization, biofilm formation, phage infectivity, bacterial resistance, and interaction with the host (Manara et al., 2018). The net charge of WTA and LTA is one of the important factors affecting the sensitivity of *S. aureus* and many other G^+^ bacteria to cationic anti-microorganism peptides (CAMPs). To reduce the highly negative net charge in the cell envelope, WTA was modified with D-Ala. Peschel et al. (Peschel et al., 1999) proposed that D-Alaized WTA is involved in mediating *S. aureus* antimicrobial peptide resistance. The *dlt*ABCD operon encodes the enzyme required for the D-Alaization of WTA. By biochemical characterization, researchers designed an inhibitor of DltA: d-alanyl-aminoaminoadenide (May et al., 2005), which could block d-Ala adenosine of DltA *in vitro*. When combined with vancomycin *in vivo*, the growth of *Bacillus subtilis* was significantly inhibited. Although this inhibitor has a high affinity for DltA of *B. subtilis*, the MIC is rather high, which may relate to its degradation in cells or limited permeability. This inhibitor also inhibits DltA in MRSA, which is also more effective when combined with *β*-lactam antibiotics, especially with imipenem (Coupri et al., 2021). In addition, D-Alanylation-deficient *E. faecalis* is more sensitive to β-lactam antibiotics (Coupri et al., 2019). D-Alaization is very important for the virulence of bacteria as well as for infection. For example, *S. aureus* lacking *dltA* are more sensitive to neutrophils and host-produced phospholipase-degrading bacterial membrane lipids (Hunt et al., 2006). Although it has been reported to inhibit *dltA in vitro* (May et al., 2005), Amsacrine is the only molecule that has been shown to inhibit D-Alaization in cells and restores D-Alaization in mutants expressing the resistant *dltB* allele. Thus, DltB is a target for Amsacrine (Pasquina et al., 2016).

The susceptibility of *S. aureus* to CAMPs and Glycopeptide antibiotics, such as Vancomycin or Teicoplanin, has increased (Brown et al., 2012), and is obviously fatal to *S. aureus*. Orivancin bound to lipid II can prevent the regeneration of the shared lipid transporter C55 that is required for the PG and WTA biosynthesis, and WTA inhibition takes precedence over PG inhibition (Singh et al., 2017). In addition, WTA β-GlcNAc glycosylation modification is essential for MRSA to maintain β-lactam resistance (Brown et al., 2012).

Park *et al* found that human mannose-binding lectin (MBL) binds to *S. aureus* cells by recognizing the cell surface sugar polymer WTA, and this binding activates the lectin complement pathway, which can induce deposition of C4 on *S. aureus*. The *S. aureus tagO* mutant lacking WTA cannot bind to purified human MBL (MBL/MASP complex), but re-introduction of the plasmid-encoded *tagO* gene can restore binding (Park et al., 2010). This makes the *tagO* gene a potential target for new antibacterial agents. The *tagO* gene, which encodes TagO, is a key enzyme that catalyzes the first step in WTA biosynthesis, and can be inhibited by a natural product, tunicamycin (Zhu et al., 2018). Knocking out the *tagO* gene, *S. aureus* is highly sensitive to autolysis (Soldo et al., 2002; Atilano et al., 2010; Schlag et al., 2010), and mutation in *tagO* increases the hydrophobicity of the bacterial membrane surface and reduce the adhesion ability (Holland et al., 2011). TagO possesses a high homology with MraY in *Bacillus subtilis*, suggesting that it may be involved in the synthetic reaction of peptidoglycans (Soldo et al., 2002). Recently, a new inhibitor of LTA, HSGN-94/189 (Naclerio et al., 2020), has been identified to have antibacterial activity on MRSA and VRE, especially when combined with tunicamycin. Opoku-Temeng et al. found that the active group in HSGN-94/189 contained N-(1,3,4-oxadiazol-2-yl) benzamide moieties and was effective in reducing bacterial loading *in vivo* (Opoku-Temeng et al., 2018). All above results suggested that HSGN-94/189 may also target *TagO*.

In addition to *TagO*, other members from the *Tag* family, *TagA*, *TagB*, *TagC,* and *TagG*, can also be treated as targets for the development of new antibiotics. TagA glycosyltransferase is involved in the first step in WTA synthesis. *tagA*-deficient strains are less toxic and sensitive to methicillin, imipenem, and ceftazidime (D'Elia et al., 2006a,b; Farha et al., 2015). Moreover, it has found that five residues (E210, W211, R214, R221, and R224) of the C-terminal in TagA are crucial to its catalysis (Martinez et al., 2022), suggesting the discovery of new antibiotics that could be achieved by disrupting the synthesis of important polymers. Similarly, the serine active sites of TagB and TagC were also discovered, which play an important role in bacterial adhesion, aggregation, invasion, and infection (Pokharel et al., 2020). Researchers found that the TagB-S255A and TagC-S252A proteins could not enter the cells or cause any cytopathy. Thus, the revelation of these active sites also provides new possibilities for the development of new antibiotics. TarG is the main component of the ABC transporter TarGH, and the novel antibiotics Targocil and 1835F03 can interact with TarG to inhibit TarGH activity (Swoboda et al., 2009; Lee et al., 2016). MnaA is a 2-isopropylase that regulates TarO and TarA through the mutual conversion of UDP-GlcNAc and UDP-ManNAc. Tunicamycin has been shown to bind to MnaA and inhibit the activity of 2-isopropylase in a dose-dependent manner (Mann et al., 2016). In addition, the UDP-GlcNAc2-isopropylase inhibitor epimerox, which was identified based on the crystal structure of *Bacillus anthracis* UDP-GlcNAc2-isopropylase, showed strong anti-*S. aureus* and anti-*S. epidermidis* activity (Schuch et al., 2013; Xu et al., 2013). But whether epimerox can inhibit WTA synthesis and restore the antibacterial activity of *β*-lactam antibiotics still needs to be further explored. All in all, as a new antibiotic target, MnaA has good development and application prospects. On one hand, it can inhibit the synthesis of WTA alone; on the other hand, it can be used as an adjuvant combined with *β*-lactam antibiotics to restore its activity to kill MRSA and MRSE.

WTA-mediated deposition of C4 in adult serum is not induced by the MBL/MASP-mediated lectin pathway, but by the classical pathway mediated by Clq. After anti-WTA-IgG (mainly IgG2) is produced, it can induce complement-dependent opsonophagocytosis against *S. aureus*. Not only that, GlcNAc residues were identified as epitopes against *staphylococcal* WTA of serum IgG and MBL, especially the γ-configuration (Kurokawa et al., 2016). This provides a new strategy for accelerating the development of new drugs and preventing MRSA infections.

**Figure 4:**
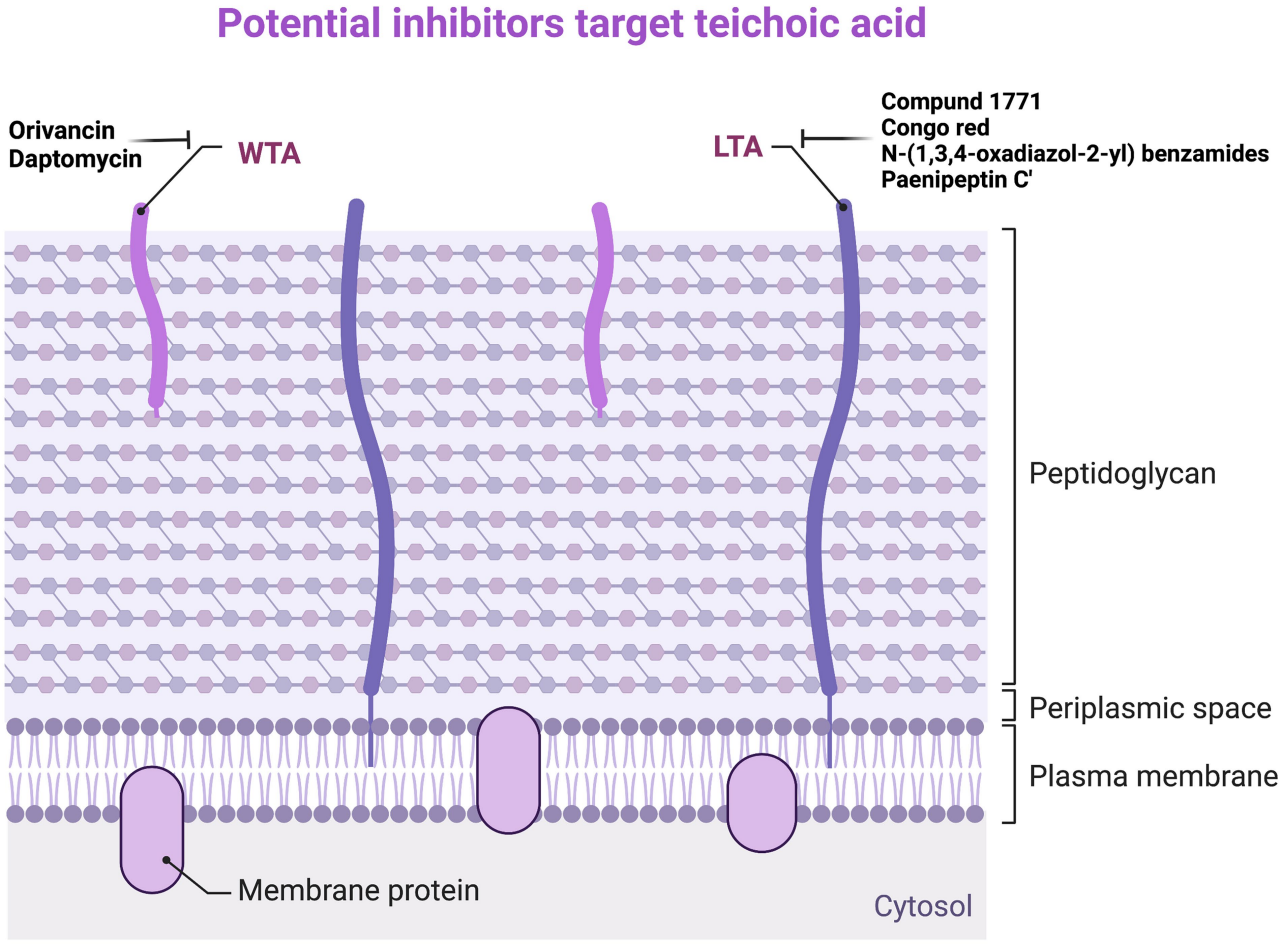
Potential inhibitors target teichoic acid. WTA, wall teichoic acid; LTA, lipoteichoic acid.

Daptomycin is a lipopeptide antibiotic active against G^+^ bacteria, which binds to the membrane in the presence of Ca^2+^ to inhibit LTA synthesis. The bactericidal activity of Daptomycin against *Staphylococci* spp. and *Enterococci spp.* is much higher than that of β-lactam antibiotics and Vancomycin. LTA is synthesized from phosphatidylglycerol, and the reaction is catalyzed by lipoprotein acid synthase (LtaS). The depletion of the *ltaS* genes and LTA in *S. aureus* can lead to growth arrest, enveloping, and cell division defects (Grundling and Schneewind, 2007). Many compounds against LTA have been found in previous studies. The first inhibitor that can inhibit LTA biosynthesis is Compound 1771, which has a MIC of 5.34 μg/ml against *S. aureus* (Richter et al., 2013). Later, Vickery et al. proved that Congo red has inhibitory activity against LtaS, but its antibacterial activity against *S. aureus* is very low, and its MIC is 1,024 μg/ml (Vickery et al., 2018). The latest study has found a new inhibitor, N-(1,3,4-oxadiazol-2-yl) benzamides, that can inhibit LTA biosynthesis, and its MIC value for MRSA is 0.25 μg/ml, which is several times stronger than Vancomycin and Linezolid (Naclerio et al., 2019). In addition, Huang et al. found that the LTA of *S. aureus* ATCC 29213 showed a dose-dependent manner to Paenipeptin C′ activity. LTA on the surface of G^+^ bacterial cells may be the original target of Paenipeptin C′. Through transmission electron microscopy, it can be observed that, after treatment with 32 μg/ml Paenipeptin C′ for 2 h, the cell wall of *S. aureus* ATCC 29213 disappeared, the cell membrane was ruptured, and the cell contents flowed out (Moon and Huang, 2019). Although it is not ruled out that Paenipeptin C′ exerts antibacterial effects by acting on certain targets within the cell, it can lead to changes in cell membrane structure, affecting the growth of bacteria and inhibiting the synthesis of LTA (Figure 4; Supplementary Table S3).

## Lipoprotein

Bacterial lipoprotein (LPP) belongs to pathogen-associated molecular patterns (PAMPs) that can regulate the host’s immune response. The maturation of LPP depends on three key enzymes, namely pro-LPP diacylglycerol transferase (LGT), pro-LPP signal peptidase II (Spase II, or lipoprotein signal peptidase, LspA), and carrier lipoprotein N-acyltransferase (LNT) (Banerjee and Sankaran, 2013). Some G-bacteria can also secrete LPPs to the extracellular environment, and these LPPs have Peptidoglycan-associated lipoprotein (Pal), which plays an important role in the pathogenesis. Thus, researchers can develop new vaccines to target these LPPs (Table 1).

**Table 1:** Potential inhibitors target lipoprotein.

Cell wall composition	Targets	Potential inhibitors
Lipoprotein	Lgt LspA	G2824 (Diao et al., 2021) Globomycin and its derivatives (Dev et al., 1985) Myxovirescin A (Gerth et al., 1982) BZM-1j and its analogues (Kitamura et al., 2018)
	MntC FhuD2 Tol-Pal OspA	Anti-MntC monoclonal antibodies Anderson et al., 2012 / Vaccines (Henry et al., 2004) OspA vaccines (Sigal et al., 1998; Steere et al., 1998)

Lgt is the first key enzyme involved in LPP biosynthesis, and is responsible for recognizing the motif of the lipid box and catalyzing the transfer of the diacylglycerol group from phosphatidylglycerol to the thiol group of the conserved cysteine in the forward LPP (Sankaran and Wu, 1994). The deletion of the *lgt* gene is fatal to the growth of G^+^ bacteria such as *Streptomyces coelicolor* (Thompson et al., 2010) and *M. tuberculosis* (Tschumi et al., 2012), as well as certain G-bacteria (Chimalapati et al., 2012). For example, the deletion of the *lgt* gene of *Listeria monocytogenes* not only inhibits the growth of bacteria but may also weaken the virulence by changing the extracellular LPP level (Reglier-Poupet et al., 2003; Baumgartner et al., 2007). Although the lack of Lgt may not necessarily prevent the growth of bacteria, it will greatly affect the performance, function, and virulence of the LPP surface (Chimalapati et al., 2012). By analyzing the X-ray crystal structure of the Lgt of *E. coli* and the Phosphatidylglycerol/Palmitic acid complex, researchers found that the hydrophobic tail of palmitic acid extends deeply into a narrow hydrophobic region, while the head domain is negatively charged. Lgt forms a salt bridge with R143 (one of the most important residues for the transfer of diacylglycerol groups), which happens to be in the least hydrophobic region. Although Palmitic acid can inhibit Lgt non-specifically (Mao et al., 2016), no specific inhibitor of Lgt has been found so far. In the future, inhibitors of Lgt can be designed based on key amino acid residues and structural characteristics of the catalytic chamber. Recently, G2824 was identified to inhibit bacterial cell growth by inhibiting the diacylglyceroltransferase activity of Lgt (Diao et al., 2021). However, whether G2824 works by acting on the residues and/or catalytically active chambers still needs further studies.

LspA is encoded by the *lsp* gene, which is present in G-bacteria, G^+^ bacteria, and mycoplasma but not in archaea and eukaryotes (Paetzel et al., 2002). Most bacteria have only one *lsp* gene (Kovacs-Simon et al., 2011; Nakayama et al., 2012). Multiple studies on *lsp* gene knockout have shown that LspA plays a vital role in the growth, function, and virulence of bacteria. In *S. pneumoniae* infection animal models, the loss of LspA leads to the accumulation of immature LPP and the attachment to the cell surface, impairing the function of the ABC transporter and reducing bacterial replication (Khandavilli et al., 2008). For *S. coelicolor*, the lack of LspA causes the loss of LPPs on the cell membrane, leading to developmental defects (Munnoch et al., 2016). So far, two types of LspA specific inhibitors have been reported, including (1) antibiotics originally identified from natural products, namely Coccinomycin and its derivatives (Dev et al., 1985), and Myxovirescin A (or antibiotic TA; Gerth et al., 1982), and (2) small molecule compounds, namely BZM-1j and its analogues (Kitamura et al., 2018).

Lnt catalyzes the transfer of the sn-1 acyl chain of the phospholipid to the α-amino group of apolipoproteins lipidated cysteine (i.e., diacyl lipoprotein; Jackowski and Rock, 1986; Gupta and Wu, 1991), with the widest optimal pH range of 6.5 to 7.5 (Gupta and Wu, 1991). Lnt is mainly distributed in G-bacteria such as *E. coli* and G^+^ bacteria with high GC content, such as *Mycobacterium* spp. and *Streptomyces spp*. Although Triacylated lipoproteins have been identified in some categories, i.e., *S. aureus* and *B. subtilis*, no true Lnt homologs in G^+^ bacteria with low acyl content (i.e., Firmicutes) have been confirmed (Kurokawa et al., 2012; Nguyen and Gotz, 2016). Although the structure of Lnt was determined by X-ray, no specific inhibitor has been found so far (Vidal-Ingigliardi et al., 2007; Buddelmeijer and Young, 2010).

Peptidoglycan-associated lipoprotein (Pal) is very important for the pathogenic mechanism and survival of G-bacteria. Pal is anchored in the OM and interacts with the Tol protein to form the Tol-Pal complex. The Tol-Pal complex consists of five proteins: TolQ, TolR, TolA, TolB, and Pal. Mutations in the *tol-pal* gene can cause various physiological changes in bacteria. *E. coli* mutants; for example, *tolA*, *tolQ*, *tolR,* and *pal* can produce a large number of outer membrane vesicles (OMV; Bernadac et al., 1998; Balsalobre et al., 2006), which are secreted to the outside of the cell, and may have promising prospects in antibacterial aspects as they can be used as carriers to deliver antibiotics to eukaryotic cells or for vaccine development (Henry et al., 2004; Ferrari et al., 2006). The Tol-Pal protein is not only related to the secretion of OMV, but also participates in the formation of cell membranes in daughter cells. A study has found that the Tol-Pal protein accumulates at the site of cell contraction, forming a membrane that separates daughter cells (Gerding et al., 2007), and plays a role in the absorption/transportation of certain compounds in the plasma membrane (Llamas et al., 2003). The *tol*QRA gene mutant of *Vibrio cholerae* created by Heilpern & Waldor (Heilpern and Waldor, 2000) has defects in transporting the bacteriophage CTXΦ DNA through the periplasm, which reduces the mutant’s ability for infection, resulting in a decrease in the secretion of cholera exotoxin and reducing the symptoms of diarrhea. The addition of purified 10 mg of Pal to the C3H/HeJ mouse cell line resulted in the induction of immune responses in macrophages and splenic lymphocytes like those observed after LPS application. TNF-α, IL-6, and nitric oxide are all increased, and early sepsis symptoms are induced (Yamauchi et al., 2006). The survival rate of C3H/HeN mice infected by *E. coli* strains with reduced expression of the *pal* gene increased and the level of IL-6 decreased (Hellman et al., 2002; Liang et al., 2005). The skin infection caused by the *Haemophilus ducreyi pal* mutant is not very serious, and it rarely transforms into ulcer form compared to the wild-type strain, which may be due to the reduced survival rate or the ability to induce immune response and the recruitment of neutrophils, lymphocytes, macrophages, and dendritic cells to the infection site. Thus, fewer *H. dukkerii* are isolated from the biopsy sample of the infection site (Fortney et al., 2000).

The Tol-Pal protein system can be used for the development of new vaccines and a good carrier for delivering antibiotics into eukaryotic cells. For example, *Neisseria meningitidis* has four serogroups A, C, W-135, and Y, that cause sepsis and meningitis. Effective vaccines based on conjugated capsular polysaccharides have been developed to prevent their infections. For type B serogroups, the anti-MenB vaccine based on OMV containing OM protein has been tested in phase III clinical trials with promising results (Henry et al., 2004; O'Hallahan et al., 2004; Ferrari et al., 2006). Yoon et al. (2002) developed a DNA vaccine based on the *Legionella pneumophila’s pal* gene and found intramuscular administration of a plasmid expressing the *pal* gene with eukaryotic promoters can induce a Th1 type response and a strong cytotoxic response. This shows that the DNA encoding the *pal* gene vaccines have great prospects as vaccines against Legionnaires’ disease.

MntC is also a potential target which has an impact on virulence. According to some studies, the toxicity of pro-Lpp lipidation deficient *S. aureus* mutants (*slgt*) is significantly decreased, particularly for iron malabsorption (Schmaler et al., 2009). MntC is also called SitC because it is involved in the transportation of iron (Cockayne et al., 1998; Muller et al., 2010). In the serum of the mouse host during the infection and recovery phase, most of the surface-related proteins expressed in the body are LPPs involved in nutrient absorption and metal ion acquisition. However, only MntC is the manganese-binding protein of the MntABC system, which is essential for the virulence of MRSA during systemic infection in mice (Kehl-Fie et al., 2013). It can be observed that active immunization with MntC can reduce the bacterial load in *S. aureus* and *S. epidermidis* infections, while anti-MntC monoclonal antibodies have a protective effect in the passive immunization model that induces neutrophil respiratory burst (Anderson et al., 2012). In *Staphylococcus*, MntC is a highly conserved sequence, while MntC and SitA from *S. aureus* are orthologous. The sequence has a high degree of identity and has been well obtained in biochemistry and structure characterization (Gribenko et al., 2013; Abate et al., 2014). They may be the best candidates for anti-*Staphylococcus* vaccines.

Clinical trials in the United States have shown that Lyme disease can be prevented by inoculation with OspA, which is the major surface LPP encoded by all *Borrelia burgdorferi* (Sigal et al., 1998; Steere et al., 1998). Although there are no antibiotics against bacterial LPPs currently in clinical use, those LPPs can be used as reliable targets for antibacterial vaccine screening and the development of new antibacterial drugs.

## Other inhibitors

### Nanoparticles

With the extensive research, the application of nanotechnology has been promoted, and many nano-molecules or nano-compounds are now available. Some of these possess potential application value in the field of medical and health care (Thabit et al., 2015). Since the antimicrobial mechanism is different from that of traditional antimicrobials, the development of NPs was treated as an alternative to solve antimicrobial resistance problem (Akhtar et al., 2015). Most metal atoms are developed as nano-antimicrobial agents, including silver, copper, titanium, magnesium, and zinc (Fatima et al., 2021). Silver has been known to possess antibacterial property for a long time. Blessed with nanotechnology, its antibacterial efficacy has been further improved to treat *S. aureus, E. coli, P. aeruginosa, K. pneumoniae, Salmonella typhi, Bacillus cereus*, and *Vibrio hemolyticus* (Siddiqi et al., 2018). Compared with silver, silver nanoparticles (AgNPs) reduce in volume while greatly increasing their surface area, which can produce ROS to disrupt the cell wall (Gomaa, 2017). Copper ions, including CuSO_4_, Cu(OH)_2_ and copper polymers, have antimicrobial activity against many microorganisms, including *S. aureus*, *E. coli*, *Enteric-soluble Streptococcus* spp., and *L. monocytogenes* (Kruk et al., 2015). Meanwhile, CuNPs could induce lipid peroxidation, electrostatic interaction, protein denaturation destruction, and DNA degradation, which eventually cause bacteria death (Thekkae Padil and Cernik, 2013). Nano gold particles could target OM, with a large accumulation on the membrane surface, upregulating the production of ROS to kill *S. aureus*, *E. coli*, *K. pneumoniae*, and *B. subtilis* (Shamaila et al., 2016). In addition, after modification of its functional groups, Nano gold could possess effective inhibition against the growth of MRSA (Abdel-Kareem and Zohri, 2018). Due to photocatalytic properties, titanium NPs also could lead to ROS production, resulting in oxidation of cell components, destruction, and inhibition (Baptista et al., 2018).

As well as the development of metal atoms into nanoparticles, non-metallic elements and other substances have also developed. Carbon nanomaterials (CNSs) such as graphene, fullerene, and carbon nanotubes could induce the production of ROS to destruct cell walls (Al-Jumaili et al., 2017). ε-polylysine (ε-PL) mainly consists of 25–30 L-lysine residues connected to ε-amino groups and α-carboxyl bonds. With its non-toxic, water-soluble, and biodegradable characteristics, it is used as a drug carrier. Studies have shown that ε-polylysine NPs can disrupt the cell walls as well as chemotactic and RNA transport systems (Naghadeh et al., 2017). In addition, chitosan and haloamine NPs are also antibacterial candidates (Demir et al., 2017).

The antibacterial mechanism of NPs is mainly originated from the strong positive zeta potential, which can interact with the cell membrane, resulting in its destruction. In addition, this strong electrostatic attraction can enhance the penetration of NPs into cell membranes (Chatterjee et al., 2015). Therefore, the antibacterial activity of NPs against G-bacteria is stronger than that of G^+^ bacteria (Pazos and Peters, 2019).

### Antimicrobial peptides

Antimicrobial peptides (AMP) are components of the immune system in many organisms, such as bacteria, plants, fish, amphibians, insects, mammals, and even viruses (Papo and Shai, 2003; Hancock and Sahl, 2006; Etayash et al., 2013; Fry, 2018). AMP self-assembles on the pronuclear membrane by hydrophobic/electrostatic interactions, followed by the formation of transmembrane pores. Then, cell membrane disintegration, mitochondria leakage, and ribosome organelles disfunction leadto cell death. AMPs such as Lactococcin-G and Contococin-1,071 can interact with the *uppP* gene, inhibiting the synthesis of cell walls (Kjos et al., 2014; Belguesmia et al., 2017). AMPs can also bind to lipid-II, such as lantibiotics, disrupting the synthesis of peptidoglycans (Islam et al., 2012; Yount and Yeaman, 2013). In addition, there are AMPs (e.g., Ѳ-defensin) that could induce certain enzymes (e.g., N-acetyl-alanine amidase) to cause cell wall disintegration (Bierbaum and Sahl, 1987; Wilmes et al., 2014; Wilmes and Sahl, 2014).

Notably, some bacteria are resistant to AMPs (Hankins et al., 2012; Malanovic and Lohner, 2016), i.e., some G-strains could exhibit resistance to AMPs by altering the acylation of lipid A units with amino arabinose (Gunn, 2001); meanwhile, other bacteria could release positively charged proteins bound to the membrane or secreted the extracellular negatively/positively charged proteins, reducing the electrostatic effect (Cullen et al., 2015). Therefore, we can indirectly screen effective inhibitors from these known drug resistance targets. Although AMP exhibits good antibacterial activity, it is rapidly degraded in the circulatory system after intravenous injection, and even deposited in the reticuloendothelial system. This could not only cause the loss of its activity, but also produce toxicity (Singh et al., 2014; VanderVen et al., 2015). In view of these limitations, using nanomaterials as the delivery carrier, such as CM-SH-Au NM, might be a potential direction (Rai et al., 2016).

### Phytocicides

Phytocicides are compounds extracted from plants and are related to plant growth and metabolism (Molyneux et al., 2007). Certain plant ingredients are used as preservatives in meat products to prevent the growth of spoilage bacteria such as *Salmonella spp.*, *Listeria spp.*, etc. (Amarowicz et al., 2005; Leitzmann, 2016; Papuc et al., 2017). For bacterial infections, carvacrol and its isomer thymol can fight against *S. pyogenes* and *S. aureus* through targeting cell membranes (Wang et al., 2016; Khan et al., 2017). Perhaps this destructive effect is related to its phosphorus wall acid, which helped the small monoterpenoid hydrophobic compound to accumulate on the cell wall surface and react with the pore protein on the OM (Wijesundara et al., 2021). Thereby, by forming a strong polymer bond, it resulted in the decomposition of the pore protein, exerting a membrane-breaking effect (Kurekci et al., 2013; Wijesundara et al., 2021). Catechin-like substances isolated from tea leaves, such as Epigallocatechin gallate (EGCG), have been found to possess antibacterial effects, which can interact with the OM (Nakayama et al., 2013) or the peptidase of sortase A to affect several virulence-related proteins located on the cell surface (Song et al., 2017). In addition, EGCG can work synergistically with β-lactam antibiotics to kill carbapenem-resistant *A. baumannii*. Since β-lactam antibiotics exert antibacterial effects by inhibiting the synthesis of penicillin-binding proteins and peptidoglycans, EGCG may possess a similar mechanism (Lee et al., 2017). The mechanism of flavonoids, such as Quercetin, depend on the formation of complex compounds with extracellular proteins to disrupt bacterial cell membranes (Wang et al., 2018). By destroying peptidoglycan components in the cell wall, Alkaloids and Curcumin could lead to bacterial death (Cushnie et al., 2014; Tyagi et al., 2015). There are many other phytocicides that are worth exploring to screen effective inhibitors.

### Plant-derived antibacterial agents

Recently, plant-derived antibacterial agents have been increasingly recognized as potential antimicrobial agents. Not only do they possess antibacterial activity, but can also be combined with antibiotics to perform synergistic antibacterial effects (Yang et al., 2010; Lu et al., 2013). Luteolin, extracted from chrysanthemum and honeysuckle, can reduce the production of α-toxins in *S. aureus* at sub-inhibitory concentrations in a dose-dependent manner. It may reverse the *E. coli* resistance to amoxicillin by inhibiting the synthesis of proteins and peptidoglycans, reducing the activity of certain ultra-broad-spectrum β-lactamase enzymes, and altering the permeability of cell membranes (Eumkeb et al., 2012). The magnoliol and magnolia phenol which are purified from magnolia can bind to penicillin-binding proteins 2a (PBP2a) and PBP4 through molecular docking. Both of these have a high Surflex score, so they could be candidates as inhibitors of peptidoglycans (Wang et al., 2010). Thus, modification of these active ingredients may significantly enhance their antibacterial effects to treat drug-resistant bacteria.

## Conclusion

Cell wall is the first barrier of bacteria; targeting its key constituents is one of the most important antibacterial strategies. The natural antibiotics acting on PG biosynthesis prove the prospect and importance of antibacterial targets against cell walls. Studies on the structure of PG biosynthetic enzymes showed that, although the substrate binding and catalytic regions are very conserved, there is a clear difference in orientation after the ligand (substrate/inhibitor) binding domain. Thus, a better understanding of the differences will provide valuable insights for the search and rational design of specific PG inhibitors.

Besides causing septic shock syndrome, the permeability barrier created by the OM of G-bacteria makes LPS a promising antibacterial drugs screening target. Thus, molecules that show direct interaction with LPS will become viable drug candidates for antibacterial and anti-endotoxin therapy. Furthermore, the availability of the three-dimensional structure of proteins that are involved in LPS binding and signal transmission is an important advancement in sepsis treatment design. The latest crystal structure of MD2-TLR4-LPS shows that there is a new interface region between MD2-TLR4 and TLR4-LPS, with a high possibility of obtaining peptide-based LPS inhibitors. In addition, many studies in recent years have used LPS as a strategy for the development of live attenuated vaccines. This strategy is based on several biological characteristics of LPS: (1) it is essential for the survival of bacteria, providing a permeability barrier, and greatly contributing to the structural integrity of bacteria (Raetz, 1990); (2) it participates in the survival of bacteria in many hosts, contributing to the resistance of complement and bactericidal peptides, as well as the adhesion and entry of bacteria into cells (Raetz, 1990; Raetz and Whitfield, 2002); (3) it contains a PAMP that is recognized by receptors on body fluids (Rietschel et al., 1994; Snyder et al., 1999); and (4) it triggers a specific antibody response.

The WTA is an important structure for the interaction between G^+^ bacteria and the host (van Dalen et al., 2020). The identified molecular interactions encompass a range of resident and circulating immune cells, epithelial and endothelial cells, and humoral immune components (antibodies and MBL). The functional consequences of the interaction between WTA and these host factors are diverse and often not fully understood. Some of them have antibacterial effects, while others may be beneficial to the survival of bacteria during infection or colonization. WTA is a promising target for preventive or therapeutic interventions against *S. aureus* (van Dalen et al., 2020), such as monoclonal antibodies, phage therapy, and vaccines. However, the heterogeneity of WTA and the adjustment of the incomplete understanding of WTA structure are both challenges in basic and translational research.

As the first enzyme of LPP biosynthesis, whether Lgt is an ideal target for drug discovery is controversial. On the one hand, inhibition of lipidation affects the positioning and function of bacterial LPPs and therefore may inhibit the growth of certain bacterial species. On the other hand, due to the link between lipidation and toxicity, it may impair the host’s recognition of pathogens (Sutcliffe et al., 2012). Screening for drug-like and effective antibacterial agents targeting LspA is still a difficult task. Coccinomycin and its derivatives lack sufficient antibacterial efficacy and are potentially toxic to the host cells. Metabolism of Myxovirescin *in vitro* is unstable, so it is not like a drug. The newly discovered Benzamide seems to show better drug likelihood, but it is not active against bacteria when used alone (Kitamura et al., 2018). So far, although the structure–function relationship of Lnt has been intensively studied (Gelis-Jeanvoine et al., 2015; Lu et al., 2017; Wiktor et al., 2017), it has not yet been targeted. Thus, more effort is needed to validate it as a suitable target.

### Future direction

In recent years, although we have actively screened for new antibacterial inhibitors, the application of these novel compounds into antibiotics is still lacking. These compounds must not only inhibit the activities of key enzymes in cell wall, but also possess antibacterial activities. One of the most successful bacterial strategies to manage the presence of antibiotics is to produce enzymes that inactivate the drug by adding specific chemical fractions to the compound that destroy the molecule itself. This renders the antibiotic unable to interact with its target. The major mechanism of β-lactam resistance depends on the destruction of these compounds by the action of β-lactamases. The damaging bond of the BLACTAM ring is destroyed by these enzymes, providing ineffective antimicrobials. Through molecular docking, the enzyme’s (ESBLs) inhibitors can be designed. In addition, a more stringent requirement is that these compounds must have molecular specificity. It is very tricky to guarantee the antibacterial activity and specificity of these inhibitors at the same time. Analogs can be developed based on existing antibiotics. However, compounds with a single enzyme target can easily lead to the production of drug-resistant microorganisms. Therefore, we can preferentially select inhibitors with multiple targets. Secondly, key functional groups can be used to modify analogue compounds. The substitution modification of key groups may greatly enhance the antibacterial effect and allow these compounds to act fully on the bacterial surface or even enter the bacteria. For example, the functional group modification allows the bacteria to promote the uptake by the bacteria through an active transport mechanism [161]. Finally, inhibitors can be screened based on compound libraries. Analyzing the structure of the natural products previously discovered, screening the rate-limiting enzymes that may possess synergistic inhibitory effects with inefficient inhibitors, or restoring the discovery of natural products through innovative targeted whole-cell screening and combination with new technologies to engineering new inhibitors are highly promising (DeVito et al., 2002; Forsyth et al., 2002).

## Author contributions

LZ and JW contributed to conception and design of the manuscript. JZ, YL, and YC wrote the first draft of the manuscript. MA, HA, and KD wrote sections of the manuscript. All authors contributed to manuscript revision, read, and approved the submitted version.

## Funding

We are grateful for the support of the National Natural Science Foundation of China (32170191 and 81903105), the Natural Science Foundation of Hubei Province (2021CFB497), and the Health Commission of Hubei Province Foundation (WJ2019H528).

## Conflict of interest

The authors declare that the research was conducted in the absence of any commercial or financial relationships that could be construed as a potential conflict of interest.

## Publisher’s note

All claims expressed in this article are solely those of the authors and do not necessarily represent those of their affiliated organizations, or those of the publisher, the editors and the reviewers. Any product that may be evaluated in this article, or claim that may be made by its manufacturer, is not guaranteed or endorsed by the publisher.
