# Supplementary material for: Breaking down the cell wall: Still an attractive antibacterial strategy
Source: Front Microbiol. 2022 Sep 23;13:952633. doi: 10.3389/fmicb.2022.952633 (PMC9544107; doi:10.3389/fmicb.2022.952633)
Supplement: Supplementary file 1 [file Table_1.DOCX]

**Table S1. Summary of potential inhibitors target peptidoglycan**

| **Composition** | **Targets** | **Potential inhibitors** |
| --- | --- | --- |
| **Peptidoglycan** | **MurA**  **MurB**  **MurC**  **MurD**  **MurE**  **MurF**  **Alr**  **Ddl**  **MurI**  **MraY**  **MurG** | Fosfomycin [262]  4-Thiazolidinones [30]  Compounds with similar structures of thiazolyl urea [31]  Sulfadoxine [28]  Pyrimethamine [28]  3,5-Dioxopyrazolidines [32]  Compound C-1 [38]  Peptides (cyclic nonapeptides:  Cys-Pro-Ala-His-Trp-Pro-His-Pro-Cys  Cys-Ser-Ala-Trp-Ser-Asn-Lys-Phe-Cys) [39]  Non-peptide inhibitors:  Phosphonates [40]  Sulfonamides [41, 42]  5-benzyliminoazoalkyl-2, 4-dione [43]  5-benzylidene mantaneamine [43]  5-benzylidenethiazolidin-4-one [45]  β-sulfonamido peptide [47]  Phosphonates [40]  Benzene 1,3-dicarboxylic acid [48]  Vancomycin [49]  Lead molecules [49]  Lifitegrast [28]  Sildenafil [28]  Sulfonamide [52]  Thiazolylaminopyrimidine series [53]  NSC 209931 [55]  8-hydroxyquinoline [56]  4-phenylpiperidine derivatives [56]  Diarylquinoline [54]  Alaphosphin [63]  Cycloserine [62]  Cycloserine [62]  D-Glu analogs [67]  Pyrazolopyrimidine dione [68]  Pyridiazepines [69]  Nucleoside antibacterial family (tunicamycin, moreomycin, carpramycin, etc.) [71]  The antibiotic E protein of phage φX174 [75]  Vancomycin derivatives containing N-chlorobiphenyl-N-methylleucine [89]  Monomycin [90]  Murgocil [91] |
|  | | |

**Table S2. Summary of potential inhibitors target lipopolysaccharide**

| **Cell wall composition** | **Targets** | **Potential inhibitors** |
| --- | --- | --- |
| **LPS** | Lipid A | E5564 [108]  Polymyxin B [113] |
|  | BamA | Darobactin [114] |
|  | PppGpp & ppGpp  MsbA | Aminoglycoside antibiotics [115, 116] (Gentamicin, Tobramycin, Amikacin)  Quinoline compounds [122] (such as G592, G913, G332) |

**Table S3. Summary of potential inhibitors target teichoic acid**

| **Cell wall composition** | **Targets** | **Potential inhibitors** |
| --- | --- | --- |
| **Teichoic acid** | **WTA**  DltA  DltB  TagO  TagA  TagB  TagC  TarG  MnaA  GlcNAc esidues  **LTA** | Orivancin [134]  d-alanyl-aminoaminoadenide [128]  Amsacrine [132]  Tunicamycin [136]  HSGN-94/189 [141]  Targocil [148]  1835F03 [149]  Tunicamycin [150]  UDP-GlcNAc2-isopropylase inhibitor epimerox [151, 152]  Daptomycin [154]  Compound 1771 [155]  Congo red [156]  N-(1,3,4-oxadiazol-2-yl) benzamides [157]  Paenipeptin C' [158] |
